# Appropriate disclosure of a diagnosis of dementia: identifying the key behaviours of 'best practice'

**DOI:** 10.1186/1472-6963-8-95

**Published:** 2008-05-01

**Authors:** Jan Lecouturier, Claire Bamford, Julian C Hughes, Jillian J Francis, Robbie Foy, Marie Johnston, Martin P Eccles

**Affiliations:** 1 Institute of Health and Society, Newcastle University, The Medical School, Framlington Place, Newcastle upon Tyne, UK; 2 Institute of Health and Society, Newcastle University, 21 Claremont Place, Newcastle upon Tyne, UK; 3 Northumbria Healthcare NHS Foundation Trust and the Institute for Ageing and Health, Newcastle University, Newcastle upon Tyne, UK; 4 Health Services Research Unit, Health Sciences Building, University of Aberdeen, Foresterhill, Aberdeen, UK; 5 School of Psychology, College of Life Sciences and Medicine, William Guild Building, University of Aberdeen, UK

## Abstract

**Background:**

Despite growing evidence that many people with dementia want to know their diagnosis, there is wide variation in attitudes of professionals towards disclosure. The disclosure of the diagnosis of dementia is increasingly recognised as being a process rather than a one-off behaviour. However, the different behaviours that contribute to this process have not been comprehensively defined. No intervention studies to improve diagnostic disclosure in dementia have been reported to date. As part of a larger study to develop an intervention to promote appropriate disclosure, we sought to identify important disclosure behaviours and explore whether supplementing a literature review with other methods would result in the identification of new behaviours.

**Methods:**

To identify a comprehensive list of behaviours in disclosure we conducted a literature review, interviewed people with dementia and informal carers, and used a consensus process involving health and social care professionals. Content analysis of the full list of behaviours was carried out.

**Results:**

Interviews were conducted with four people with dementia and six informal carers. Eight health and social care professionals took part in the consensus panel. From the interviews, consensus panel and literature review 220 behaviours were elicited, with 109 behaviours over-lapping. The interviews and consensus panel elicited 27 behaviours supplementary to the review. Those from the interviews appeared to be self-evident but highlighted deficiencies in current practice and from the panel focused largely on balancing the needs of people with dementia and family members. Behaviours were grouped into eight categories: preparing for disclosure; integrating family members; exploring the patient's perspective; disclosing the diagnosis; responding to patient reactions; focusing on quality of life and well-being; planning for the future; and communicating effectively.

**Conclusion:**

This exercise has highlighted the complexity of the process of disclosing a diagnosis of dementia in an appropriate manner. It confirms that many of the behaviours identified in the literature (often based on professional opinion rather than empirical evidence) also resonate with people with dementia and informal carers. The presence of contradictory behaviours emphasises the need to tailor the process of disclosure to individual patients and carers. Our combined methods may be relevant to other efforts to identify and define complex clinical practices for further study.

## Background

There is growing evidence that many people with dementia want to know their diagnosis [1-5]. Older people in community and hospital contexts also have positive views towards disclosure in the event of developing a dementia [6-10]. Current practice continues to lag behind this empirical evidence of preference for disclosure [11-13]. There is wide variation in the attitudes of professionals towards disclosing a diagnosis of dementia to patients [11,12]. Some practitioners acknowledge that they avoid using terms such as 'dementia' or 'Alzheimer's disease' during disclosure [14-17] and this reticence is confirmed in carer reports of disclosure. The presence of cognitive impairment demands greater attention to the repetition of information and checking of understanding. However, there is evidence that very little time is spent on elaborating or explaining the diagnosis [16]. This is reflected in poor retention of the information, with one study reporting that the majority of people with dementia (73%) and a significant minority of carers (16%) being unable to report the diagnosis accurately shortly after disclosure [18]. Even when the name of the illness is retained, the diagnosis does not necessarily help people with dementia and family members to understand and make sense of their experiences [4,16,19,20]. Uncertainty about a diagnosis is problematic not only because many people would prefer clarity, but also because uncertainty makes it difficult for people with dementia and their carers to discuss and plan for the future. Optimal management of the condition and adherence to a treatment regimen are also at risk when people are unclear about the diagnosis and its implications.

While negative reactions to disclosure, such as depression, loss of hope, psychological distress and suicide [17,21] have been cited as reasons for withholding the diagnosis, catastrophic reactions are rare [22,23]. These potentially negative consequences are offset by a range of positive consequences and many people with dementia are able to cope with their diagnosis particularly when adequate support is available [24-27]. Furthermore, the majority of people to whom a diagnosis of dementia was disclosed have positive attitudes to disclosure [5,28,29].

Although much previous work has implicitly viewed disclosure as a more or less simple one-off task, there is a growing recognition that disclosure is a complex process, comprising more than simply naming the illness. However, despite a range of practice guidelines advocating disclosure [11], there is limited specific guidance on how best to disclose a diagnosis of dementia. It has been suggested that disclosure of dementia should involve pre-diagnostic counselling or advance directives to ascertain preferences towards disclosure [9,26,30,31] and post-diagnostic interventions to facilitate absorption of and adjustment to the information [30-36]. A model for a disclosure meeting has been developed in the Netherlands which draws on existing literature on breaking bad news [32].

No intervention studies to improve diagnostic disclosure in dementia have been reported to date. As a first step in a larger study to develop a theory based intervention for health care professionals to promote disclosure [37], we sought to identify the key components of the process which could be targeted and incorporated into outcome measurements. We also explored whether supplementing a literature review with other methods would result in the identification of new disclosure behaviours. The objective was to identify the range of disclosure behaviours using three different methods.

## Methods

### Identifying behaviours

We used three methods to identify the comprehensive list of behaviours: literature review; interviews with people with dementia and informal carers; and a consensus panel process. Approval for this study was obtained from the Multi-centre Research Ethics Committee for Scotland (Reference MREC04/10/31).

### Literature review

The literature review focused on empirical and opinion-based works around breaking bad news with respect to a range of clinical conditions, including dementia but also looking at other conditions such as cancer. Four electronic databases were searched to July 2004 using the search terms shown in Table 1. The electronic search was supplemented with the reference lists of identified papers, reviews and opinion pieces. We also drew on literature regarding communication with people with dementia.

**Table 1:** Literature search

***Electronic databases searched :***
Medline 1966 to 2004
CINAHL 1982 to 2004
Web of Science 1970 to 2004
PsycInfo 1960 to 2004

***Search terms used in all databases:***
Diagnos* AND
Disclos* OR tell* OR told OR shared OR sharing OR inform* (NOT information) OR communicat* (NOT communication) OR bad news

### Interviews

In-depth face-to-face interviews were conducted with people with dementia and their informal carers recruited from two local geriatric psychiatry teams. In the interviews we sought examples of good practice and suggestions for improving the process of diagnostic disclosure. The interviews were recorded and transcribed verbatim. From five of the transcripts two researchers (JL and CB) independently developed a thematic framework. A consensus was reached on the final framework after discussion. All transcripts were subsequently coded using this framework and the data recorded in a matrix.

### Consensus Panel

The consensus panel process involved an initial postal questionnaire survey followed by a meeting conducted using the nominal group technique [38].

#### Recruitment of panel members

Health and social care professionals representing the range of disciplines involved in disclosing a diagnosis of dementia were identified and approached by the research team. Ten professionals agreed to take part in the panel: three geriatric psychiatrists, one clinical psychologist, one social worker, two general practitioners, one community psychiatric nurse, one carer support worker and one day hospital ward manager.

#### Initial postal questionnaire survey

Panellists were sent a postal questionnaire approximately three weeks before the formal meeting and asked to return it prior to the meeting. The questionnaire used open-ended questions to generate a list of possible components of, and factors influencing, disclosure. Responses to the initial questionnaire were independently categorised by two researchers (RF and JL). A summary of components and factors was produced, including the frequencies with which each item was suggested. These were presented at the structured meeting.

#### Structured meeting

At the meeting the panellists were asked first to review the components and factors identified and then to perform a ranking exercise. This was achieved through the following process:

• feedback of collated responses from initial questionnaire;

• clarification of ambiguities and areas of overlap; and

• discussion of the importance of each component and factor.

Discussion was structured to give each panellist an opportunity to comment without interruption. The research team recorded the key points made, and the moderator summarised the discussion relating to each item. A second round of comments was sought with respect to any new issues that had been raised during the first round or where panellists wished to add to their previous responses.

### Content analysis

The lists of behaviours identified by each method were compared. Duplicate behaviours were removed and a list of unique behaviours produced. An iterative process of developing mutually exclusive categories of behaviours in disclosure, and of mapping individual behaviours to these categories, was then performed jointly by two authors (CB and JL). The lists of behaviours in each category were subsequently reviewed by the entire project team. The process by which key behaviours were subsequently selected for the intervention is described elsewhere [39].

## Results

### Literature review

The literature search identified 293 articles of which 89 were considered relevant to the process of disclosure. A further 19 papers were identified from the reference lists of retrieved articles. The 108 articles included opinion pieces, reviews and original research. Each article was examined and a total of 199 components of appropriate disclosure were identified. Only two articles reporting strategies for appropriate disclosure were based on empirical data and had been evaluated.

### Interviews

We conducted 10 face-to-face interviews: four with people with dementia (in two of these the informal carer was present), and six with informal carers only. In analysing the interview data we included both explicit and implied behaviours. For example in the following quote we identified behaviours relating to 'Prepare the patient for disclosure', 'Discuss prognosis', 'Explore the patient's emotional response', 'Foster hope' and 'Explore coping strategies'.

'It's just a big shock and it's, and all I can do now is just (*pause*). I want, what's going to happen? .... I know fine well it's started, ... and I'm just dreading it, I really am dreading it.' (Patient interview 24)

Analysis of the interview transcripts identified 112 behaviours.

### Consensus panel

Eight panellists (two geriatric psychiatrists, one clinical psychologist, one social worker, two general practitioners, one community psychiatric nurse and one carer support worker) completed the initial questionnaire from which we identified 55 behaviours.

### Content analysis

Eight distinct categories of behaviours were identified: preparing for disclosure; integrating family members; exploring the patient's perspective; disclosing the diagnosis; responding to patient reactions; focusing on quality of life and well-being; planning for the future; and communicating effectively (Table 2).

**Table 2:** Summary of disclosure behaviours

	**Category (number of behaviours)**	**Sub-categories (number of behaviours)**
**1**	**Preparing for disclosure (31)**	• Plan disclosure meeting (14)
		• Arrange post-diagnosis support (2)
		• Establish rapport (3)
		• Prepare the patient (4)
		• Elicit preferences for disclosure (8)
**2**	**Integrating family members (10)**	• Identify & involve appropriate family members (4)
		• Manage differing information needs of patient & family (2)
		• Avoid collusion with family members (4)
**3**	**Exploring the patient's perspective (13)**	• Explore patient ideas (11)
		• Elicit patient expectations (2)
**4**	**Disclosing the diagnosis (33)**	• Tailor information to patient preferences & ideas (7)
		• Check understanding (7)
		• Explore the meaning(s) of the diagnosis (12)
		• Discuss prognosis (7)
**5**	**Responding to patient's reactions (24)**	• Explore the patient's emotional response (11)
		• Elicit & address patient questions & concerns (13)
**6**	**Focusing on quality of life & well-being (17)**	• Foster hope (9)
		• Explore coping strategies (8)
**7**	**Planning for the future (41)**	• Clarify follow up arrangements (8)
		• Discuss support services available (7)
		• Negotiate management plan (19)
		• Discuss prevention & health promotion (7)
**8**	**Communicating effectively (51)**	• Develop rapport (5)
		• Use appropriate verbal & non-verbal communication (20)
		• Use active listening skills (5)
		• Involve the patient (8)
		• Structure & signpost the consultation (7)
		• Consider issues of anti-discriminatory practice (6)

*Preparing for disclosure *mainly concerns behaviours completed prior to the disclosure meeting. In addition to practical arrangements, it includes aspects of pre-diagnostic counselling such as establishing patient preferences for disclosure and raising the possibility that dementia may be a possible diagnosis. Planning the approach to disclosure, for example, identifying the approach most suited to the individual patient, is also included.

The behaviours comprising *Integrating family members *focus on balancing the need to ensure that family members have access to appropriate information with the need to avoid marginalising the patient or colluding with family members.

*Exploring the patient's perspective *includes behaviours that may have formed part of earlier meetings as well as the disclosure meeting. The aim of these behaviours is to reduce or manage the gap between the information to be disclosed and patient beliefs and expectations. These behaviours also allow the professional responsible for disclosure to identify an appropriate starting point.

Behaviours related to *disclosing the diagnosis *include the provision of information on prognosis as well as diagnosis. This category includes a range of behaviours concerned with checking understanding and exploring the meanings of dementia. It therefore extends beyond simply naming the disease.

*Responding to patient reactions *involves first allowing the patient space to process the information and then exploring the range of emotional reactions that may follow disclosure. This includes exploring the reasons behind the emotional reactions as a means of further understanding the patient's perspective. A second group of behaviours are related to eliciting and addressing patient questions and concerns, whilst recognising that the patient may find it difficult to articulate questions.

*Addressing quality of life and well-being *includes fostering a (realistic) sense of hope, for example, by emphasising preserved abilities and skills and avoiding excess disability by unnecessarily curtailing social activities. Additional behaviours focus on coping strategies that may be used in helping the patient to adjust to the diagnosis.

*Planning for the future *includes arrangements for liaison, referral and follow up. Whilst the development of some management strategies may have a place within a disclosure meeting, there is recognition that many decisions would be better delayed to subsequent meetings when the patient has had time to absorb the information.

*Communicating effectively *underpins the behaviours in the remaining categories. A wide range of skills are encompassed, including building rapport, listening skills, using appropriate language, structuring and signposting the consultation. This also includes a number of behaviours relating to anti-discriminatory practice.

There were a number of inconsistencies in the behaviours identified. For example, although explicitly naming the illness and avoiding euphemisms were two behaviours identified, these seemed to contradict a third behaviour of 'Using terminology carefully as a way of getting information across without telling patients what they don't want to hear'. Similarly, while the predominant emphasis was on eliciting patient preferences for disclosure to themselves and others, the issue of obtaining carer approval prior to disclosure to the patient was also identified.

### Behaviours identified by different methods

**Figure 1:**
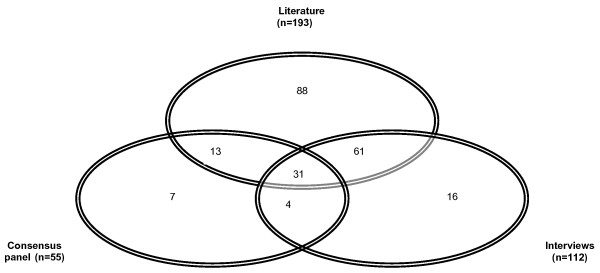
Number and overlap of behaviours identified from each source.

We examined the extent to which behaviours identified by different methods overlapped or were unique. Of the total 220 behaviours identified (see Additional file 1), 109 overlapped (Figure 1) with 31 being elicited from all three sources (Table 3). 82% of the behaviours identified from the interviews and 80% by the panel overlapped with those from the literature. The overlap was greatest in behaviours related to disclosing the diagnosis: 70% of behaviours in this category were identified from more than one source, primarily interviews and literature.

**Table 3:** Behaviours identified from all three sources

• Organise a private, quiet, comfortable location
• Schedule ample time
• Establish a trusting & supportive relationship with the patient
• Identify the most appropriate approach to disclosure based on knowledge of the patient and family
• Identify informal support available for the patient after disclosure
• Identify formal support available for the patient after disclosure
• Prepare the patient in earlier consultations
• Break the news over a series of contacts
• Discuss ahead of time how much information the patient would like about diagnosis and prognosis
• Respect the patient's right (not) to know
• Negotiate the presence of a relative with the patient
• Establish the patient's perceptions about their symptoms
• Give information step by step according to the patient's ability to cope with it
• Use terminology carefully as a way of getting information across without telling patients what they don't want to hear
• Check understanding frequently
• Be direct in disclosing the diagnosis
• Explicitly name the illness
• Avoid the use of technical terminology or medical jargon
• Discuss how the person's current problems may progress in the light of the probable diagnosis
• Provide an opportunity for the patient to absorb and emotionally process the information
• Create time and space for the individual to explore what the diagnosis means to them
• Provide an opportunity to discuss the diagnosis again, answer questions & clarify matters
• Repeat or reinforce information as required
• Document the information given and to whom
• Identify further medical and social care pathways
• Ensure information is consistent across professionals
• Provide (written) information on practical & emotional support available from health & social care services
• Provide (written) information on practical & emotional support available from voluntary organisations
• Identify the (practical) implications of the diagnosis
• Disclose all the treatment options (including no treatment)
• Do not impart too much information in one session

### Behaviours elicited from the literature only

Eighty eight behaviours were elicited only in the literature review (Figure 1). The largest proportion were behaviours associated with effective communication (41%) followed by planning for the future (17%). Few behaviours related to disclosing the diagnosis (3%) were identified exclusively in the literature.

### Behaviours elicited from the interviews only

Sixteen of the behaviours were exclusively identified by the interviews. These additional behaviours (examples given in Table 4) could be grouped into four categories relating to:

**Table 4:** Quotes illustrating behaviours identified in interviews

***Issues specific to dementia***
We've been talking really just about the dementia, haven't we, really, not the full hog of Alzheimer's.
Patient interview 21 325-6
You mean the memory? Oh, I never thought of it as an illness."
Patient interview 23 215-6

***Support needs***
It's been three years now I think, so it's been horrible and it's on my mind all the time, all the time, there's not a day goes by where I don't think about it."
Patient interview 24 109-11

***Role of carers***
"I think at the time it would have probably been me that would have needed to sort of, emphasise maybe, that he, that if, that I could be expressing things to him in a way that would help him to understand, rather than the doctors who are, th-th they only see him once, twice, you know [mmm] don't see him very often."
Carer 22 162-6
"If she's getting information there it would be nice to know what she's getting because I'm the one that does the supporting in between whiles."
Carer 23 239-41

***Need for a person-centred approach***
This doctor, he was about fifteen feet away from me, and I heard him say 'Oh, just tell him he's had a stroke and he's got Alzheimers'.
Patient interview 24 37-39

• issues specific to dementia (e.g. the need to distinguish between dementia and normal ageing);

• support needs (e.g. providing opportunities for peer support);

• the role of carers in disclosure (e.g. informing the carer what the patient has been told);

• the need for a person-centred approach (e.g. disclosing the diagnosis directly to the patient).

Several behaviours elicited in the interviews with people with dementia and carers may not have been identified in other sources because they appeared to be self-evident. For example, the need for direct disclosure to the person with dementia stemmed from experiences of three interviewees who had received the disclosure indirectly, either by overhearing professionals talking between themselves or by deducing the diagnosis from a letter from the dementia support worker at the local branch of the Alzheimer's Society. Similarly, the need for disclosure by a professional was identified by a carer who had been responsible for breaking the news to the patient.

### Behaviours elicited from the consensus panel only

Seven behaviours were elicited only by the consensus panel. Five of these behaviours were concerned with the role of family members, with behaviours focusing both on ensuring that family members' views were taken into account and their needs were met, and on maintaining a central focus on the patient and recognising the potential conflicts of interest between patients and family members.

**Figure 2:**
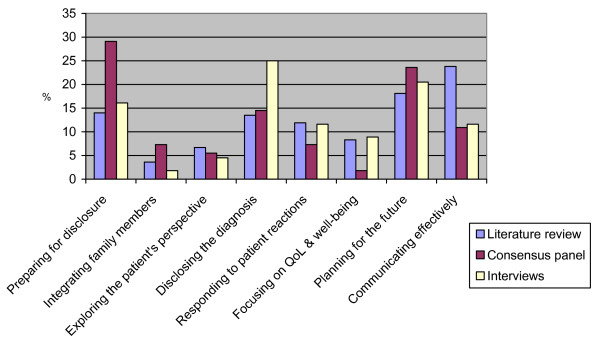
The proportion of behaviours in each category by source.

Behaviours from each of our eight categories were identified from each source, although there were differences in emphasis (Figure 2). The interviews with people with dementia and informal carers placed more emphasis on behaviours relating to disclosing the diagnosis (25%) with lower proportions of behaviours identified by the consensus panel (14.5%) and literature review (13.5%) in this category. In contrast to those identified by other sources, twice as many of the behaviours identified by the consensus panel were associated with preparing for disclosure (29.1% vs 16.1% and 14.0% of behaviours identified by the interviews and literature search respectively). The consensus panel also focused more on integrating family members (7.3%). The consensus panel identified proportionally fewer behaviours relating to focusing on quality of life and well-being than either of the other methods. The literature review placed a greater emphasis on communicating effectively (23.8% vs 11.6% and 10.9% for the interviews and consensus panel respectively).

## Discussion

This study identified key components of the process of disclosing a diagnosis of dementia as part of the development of an intervention to promote appropriate disclosure and explored whether supplementing a literature review with other methods would result in the identification of additional behaviours. The literature search identified the largest number of behaviours (193), with the panel and interviews together eliciting an additional 27 behaviours. The high level of agreement between data from the literature, interviews and consensus panel suggests that although much of the literature focuses on breaking bad news to people with cancer, it is nevertheless highly relevant to disclosing a diagnosis of dementia. The interviews not only provided behaviours unique to people with dementia and their carers but also confirmed the need for intervention by highlighting deficiencies in current practice. The panel placed a greater emphasis on preparing for disclosure and managing the role of family members in the disclosure process than was expressed in the literature.

Our eight categories of behaviours relating to diagnostic disclosure are consistent with recent studies of disclosure in dementia. The need for pre-diagnostic counselling and preparation for disclosure has been emphasised [29-31,36]. While some people with dementia and family members anticipate their diagnosis [2] others have not previously considered dementia as a possible cause for their problems [5,40]. The latter group are likely to perceive the diagnosis as a shock [2] and this may limit their ability to process the information [32]. Advising people of the potential for a diagnosis of dementia resulted in lower levels of anxiety after disclosure of the formal diagnosis [22] confirming the value of adequate preparation. Exploring the patient's perspective also enables explanations to be linked to their personal experience which may facilitate understanding [4,32]. Although some behaviours, such as holding the meeting in an appropriate physical setting and the presence of professionals already known to the person with dementia and family, may seem self-evident, their importance has also been documented in previous studies [29,32,41].

Integrating family member(s) into the process of disclosure provides an opportunity for patients and carers to learn to talk together about the diagnosis [32,42]. Given that much of the process of adjusting to a diagnosis of dementia takes place without professional involvement [1], providing a supportive social context in which people with dementia can undertake this process is essential [3,5,27,36]. The importance of opportunities to talk separately to people with dementia and family member(s) has also been highlighted, since family member(s) can find it difficult to speak openly about their difficulties and fears in the presence of the person with dementia [36,43]. Furthermore, joint meetings can lead to inadequate exploration of the patient's perspective due to the tendency of family members to speak for people with dementia [36,43].

Contradictory behaviours were identified in relation to disclosing the diagnosis. Consistent with previous literature, being direct and using explicit terminology were included [16,41]. However, the need to use terminology carefully to avoid burdening the patient with unwanted information was also identified. These contradictions emphasise the need to tailor the process (and terminology) to the preferences of individual patients and their families. Similar issues arise in relation to the discussion of prognosis. While a number of behaviours relating to prognosis were identified in the present study, preferences for detailed information about the future vary [4,40]. Consequently, eliciting preferences for information on prognosis is crucial. Even after apparent disclosure, people with dementia and family members have variable understandings of their diagnosis [1,4,16,36,44], highlighting the need to check understanding and explore the meaning(s) of dementia. Analysis of audio-recordings of disclosure meetings, however, suggested that physicians paid little attention to enhancing understanding of the diagnosis and used a variety of techniques to minimise the seriousness of the diagnosis and avoid detailed discussion [16]. This suggests a clear mismatch between professional skills and competencies and the needs of people with dementia and carers.

A wide range of types and intensity of emotional reactions to a diagnosis of dementia have been reported [29,35,36]. Professionals need be prepared to manage a range of emotional responses and provide space for these to be expressed [29,32]. Since some of the distress caused by a diagnosis of dementia relates to the negative attitudes and preconceptions about the illness, it is also important to explore these and to provide a more balanced view [45]. Many of the behaviours we identified relating to quality of life and well-being are consistent with findings of recent studies. For example, studies have emphasised the need to provide information that instils positive attitudes and hope [35], emphasises the remaining capacities [32,46] and balances hope and realism [29]. Following disclosure there may be few opportunities for people with dementia and their carers to make sense of their diagnosis [31,36,47]. Post-diagnostic counselling or follow-up meetings have been suggested [30-36]. Rather than being of a prescribed format and duration [36], a more flexible approach to follow-up is required to meet the widely varying needs and preferences of people with dementia and their families.

A range of issues relating to communication were identified which have previously been highlighted in the literature, including pacing [32]; use of non-verbal forms of communication such as diagrams or flow charts [29]; and the need to summarise information to aid recall [29].

There are limitations in our study. Firstly, because of difficulties with recruitment, we were only able to conduct four interviews with people with dementia and six interviews with carers. However, despite the small sample, we identified 112 behaviours from the interview data and were able to engage in discussions in some depth. Secondly, we were somewhat surprised, considering the range of health and social care professionals who participated, that a greater number of behaviours were not identified by the consensus panel. In retrospect we feel that the structured approach used may have restricted the responses of the panel members and resulted in a focus on what they perceived as the *most important *disclosure behaviours rather than the full range of behaviours. Although a more open-ended approach may not have resulted in the identification of additional behaviours, the congruence between behaviours identified by the panel and other sources might have been increased.

Although we identified 220 component behaviours of disclosure, there are little empirical data available concerning either the extent to which these behaviours are performed in routine practice or their influence on the experience of receiving bad news. We believe further research is needed to fully evaluate approaches to disclosing a diagnosis not only in relation to dementia but in other life threatening or life changing clinical conditions.

## Conclusion

The use of combined methods yielded an extensive range of disclosure behaviours. The considerable duplication between behaviours identified in the literature review, interviews and consensus panel, has confirmed the relevance of behaviours originating largely from other 'bad news' contexts to people with dementia, their families and the professionals who work with them. The congruence between the behaviours we have identified and those described in literature published since our review suggests that we have produced a comprehensive list of the behavioural components of appropriate disclosure of a diagnosis of dementia. The range of behaviours confirms the complexity of appropriate disclosure and highlights the importance of pre-disclosure preparation and post-disclosure management strategies and the need for disclosure to be tailored to individual people with dementia and their families. Our combined methods may be relevant to other efforts to identify and define complex clinical practices for further study.

## Competing interests

The authors declare that they have no competing interests.

## Authors' contributions

MPE, MJ, RF, CB, JJF and JCH designed the study. JL and CB conducted the literature review, the interviews, and the analysis of interview data. CB, RF and JL convened the consensus panel and facilitated the meeting. CB, MPE, JL, JJF and RF participated in the Delphi process to select the behaviours for the larger study. JL wrote the first draft which was revised by CB and then all the other members of the study team. All authors have read and approved the final manuscript.

## Pre-publication history

The pre-publication history for this paper can be accessed here:



## Supplementary Material

Additional file 1 Summary list of behaviours and sources in disclosing a diagnosis of dementia; this file lists all (220) behaviours identified and their source. Click here for file
