# Supplementary material for: Appropriate disclosure of a diagnosis of dementia: identifying the key behaviours of 'best practice'
Source: BMC Health Serv Res. 2008 May 1;8:95. doi: 10.1186/1472-6963-8-95 (PMC2408568; doi:10.1186/1472-6963-8-95)
Supplement: Additional file 1 — Summary list of behaviours and sources in disclosing a diagnosis of dementia; this file lists all (220) behaviours identified and their source. [file 1472-6963-8-95-S1.doc]

# SUMMARY LIST OF BEHAVIOURS AND SOURCES IN DISCLOSING A DIAGNOSIS OF DEMENTIA

**1. PREPARING FOR DISCLOSURE (n=31)**

|  | **BEHAVIOUR** | Literature | Consensus panel | Interviews with pwd |
| --- | --- | --- | --- | --- |
| *Plan disclosure meeting* | Organise a private, quiet, comfortable location | **✓** | **✓** | **✓** |
|  | Consider holding the disclosure meeting at the patient’s home | **✓** |  | **✓** |
|  | Schedule ample time | **✓** | **✓** | **✓** |
|  | Indicate the time available in the consultation | **✓** |  |  |
|  | Avoid interruptions / distractions | **✓** |  |  |
|  | Wait until all test results are available before giving the diagnosis | **✓** |  | **✓** |
|  | Have all information to hand | **✓** |  |  |
|  | Include other team members | **✓** | **✓** |  |
|  | Identify the most appropriate team member to disclose to individual patients (based on style, rapport) |  | **✓** | **✓** |
|  | Ensure disclosure done by a professional already known to the patient |  |  | **✓** |
|  | Ensure staff responsible for post disclosure counselling at present at the disclosure meeting | **✓** |  |  |
|  | Have clear objectives/work out an action plan before speaking to the patient | **✓** |  |  |
|  | Identify the most appropriate approach to disclosure based on knowledge of the patient & family | **✓** | **✓** | **✓** |
|  | Ensure disclosure to patient is done by a professional (not a family member or via letter) |  |  | **✓** |
| *Arrange post-diagnosis support* | Identify informal support available for the patient after disclosure | **✓** | **✓** | **✓** |
|  | Identify formal support (i.e. professional or voluntary agency) available for the patient after disclosure | **✓** | **✓** | **✓** |
| *Establish rapport* | Introduce yourself & your role | **✓** |  |  |
|  | Attend to the patient’s physical comfort | **✓** |  |  |
|  | Establish a trusting & supportive relationship with the patient | **✓** | **✓** | **✓** |
| *Prepare the patient* | Prepare the patient in earlier consultations | **✓** | **✓** | **✓** |
|  | Break the news over a series of contacts | **✓** | **✓** | **✓** |
|  | Explain that dementia may be one of the possible diagnoses in earlier consultations | **✓** |  | **✓** |
|  | Explain the process involved in establishing the diagnosis, including the nature & reason for diagnostic tests | **✓** |  | **✓** |
| *Elicit preferences re disclosure* | Discuss ahead of time how much information the patient would like about diagnosis & prognosis | **✓** | **✓** | **✓** |
|  | Avoid disclosing relevant information in attempting to ascertain patient preferences for disclosure | **✓** | **✓** |  |
|  | Respect the patient’s right (not) to know | **✓** | **✓** | **✓** |
|  | Address with the patient what information will be shared with whom | **✓** | **✓** |  |
|  | Negotiate the presence of a relative with the patient | **✓** | **✓** | **✓** |
|  | Confirm whether the patient would like a member of the family or friend to stay in the room at the time of disclosure | **✓** |  |  |
|  | Offer to tell spouse or others | **✓** |  |  |
|  | Seek family approval before disclosing to patients |  | **✓** |  |

**2. INTEGRATING FAMILY MEMBERS (n=10)**

|  | **BEHAVIOUR** | Literature | Consensus panel | Interviews with pwd |
| --- | --- | --- | --- | --- |
| *Involve appropriate family member(s)* | Encourage the patient to share the diagnosis with significant others | **✓** |  |  |
|  | Identify the most appropriate family member / ask the family to identify a spokesperson | **✓** | **✓** |  |
|  | Ensure that the people wanted by the patient are present to hear the diagnosis | **✓** |  |  |
|  | Ensure family member(s) know what the patient has been told |  |  | **✓** |
| *Manage differing information needs of patient & family members* | Arrange to see the patient & family member(s) together & individually to facilitate disclosure of difficult issues & different information needs | **✓** |  | **✓** |
|  | Consider the potential consequences of disclosure to family members |  | **✓** |  |
| *Avoid collusion with family member(s)* | Avoid collusion by disclosing to the patient first or to the patient & family members together | **✓** |  |  |
|  | Assess the reason(s) for any requests to withhold information from the patient | **✓** |  |  |
|  | Show appropriate respect for (legal issues relating to) patient confidentiality | **✓** | **✓** |  |
|  | Recognise potential conflicts of interest between patients & family members |  | **✓** |  |

**3. EXPLORING THE PATIENT’S PERSPECTIVE (n=13)**

|  | **BEHAVIOUR** | Literature | Consensus panel | Interviews with pwd |
| --- | --- | --- | --- | --- |
| *Explore patient ideas* | Establish the patient’s perceptions about their symptoms | **✓** | **✓** | **✓** |
|  | Identify cultural customs & beliefs about the illness & negotiate differences with respect | **✓** |  |  |
|  | Explore what prompted the patient to ask for assessment | **✓** |  |  |
|  | Discuss the symptoms & changes the patient & family have noticed | **✓** |  | **✓** |
|  | Ask if s/he has thought what the diagnosis might be | **✓** |  | **✓** |
|  | Find out what the patient already knows | **✓** | **✓** |  |
|  | Clarify the patient’s understanding of past tests & what they mean | **✓** |  |  |
|  | Check the strength of their beliefs | **✓** |  |  |
|  | Find out where the patient stands at each visit; often patients tend to forget information even if they seemed to fully understand it when first told | **✓** |  | **✓** |
|  | Acknowledge patient’s views & feelings without being judgemental | **✓** |  | **✓** |
|  | Identify the appropriate starting point based on the patient’s current understanding | **✓** |  |  |
| *Elicit patient expectations* | Elicit patient expectations of the consultation | **✓** | **✓** |  |
|  | Negotiate an agenda taking patient, family member(s) & clinician needs into account | **✓** |  |  |

**4. DISCLOSING THE DIAGNOSIS (n=33)**

|  | **BEHAVIOUR** | Literature | Consensus panel | Interviews with pwd |
| --- | --- | --- | --- | --- |
| *Tailor information to patient preferences & ideas* | Give information step by step according to the patient’s ability to cope with it | **✓** | **✓** | **✓** |
|  | Allow the patient to choose when they have heard enough | **✓** |  | **✓** |
|  | Prepare the patient to receive serious news – fire a warning shot | **✓** |  | **✓** |
|  | Relate the explanation to the patient’s ideas, concerns & expectations | **✓** |  |  |
|  | Use terminology carefully as a way of getting information across without telling patients what they don’t want to hear | **✓** | **✓** | **✓** |
|  | Recognise that different family members will have different information needs |  | **✓** |  |
|  | Avoid making assumptions about what patients & carers already know (e.g. based on job or previous experience) |  |  | **✓** |
| *Check understanding* | Check understanding frequently | **✓** | **✓** | **✓** |
|  | Reinforce areas that patients perceive correctly & gently educate in areas of misunderstanding | **✓** |  |  |
|  | Ask the patient to summarise what s/he understands | **✓** |  |  |
|  | Draw on the patient’s experiences, those of acquaintances or knowledge from the media, to gauge understanding | **✓** |  | **✓** |
|  | Look for a window on denial to give the patient a chance to review the situation without forcing them to accept reality when they are not ready to do so | **✓** |  | **✓** |
|  | Find out patient’s knowledge level about the disease | **✓** |  | **✓** |
|  | Help the patient understand what the diagnosis means |  |  | **✓** |
| *Explore the meaning(s) of the diagnosis* | Be honest (about the severity of the condition) | **✓** |  | **✓** |
|  | Give detailed information about the results of medical tests | **✓** |  | **✓** |
|  | Provide supporting evidence for diagnosis | **✓** |  | **✓** |
|  | Be direct in disclosing the diagnosis | **✓** | **✓** | **✓** |
|  | Explicitly name the illness | **✓** | **✓** | **✓** |
|  | Provide information on different types of dementia | **✓** |  | **✓** |
|  | Discuss the particular disease process that is causing the dementia | **✓** |  | **✓** |
|  | Give an explanation as to why the changes are happening | **✓** |  | **✓** |
|  | Give the rationale for making the diagnosis | **✓** |  | **✓** |
|  | Acknowledge any diagnostic uncertainty |  | **✓** |  |
|  | Explain the relationship between dementia & Alzheimer’s disease |  |  | **✓** |
|  | Explain the difference between normal ageing & dementia |  |  | **✓** |
| *Discuss prognosis* | Discuss how the person’s current problems may progress in the light of the probable diagnosis | **✓** | **✓** | **✓** |
|  | Describe the behavioural & psychiatric symptoms of dementia | **✓** |  | **✓** |
|  | Explain that individuals are affected differently so there is no exact way to determine progression | **✓** |  | **✓** |
|  | Give a broad timeframe for what may lie ahead | **✓** |  | **✓** |
|  | Explain that there is no curative therapy | **✓** |  | **✓** |
|  | Discuss life expectancy | **✓** |  | **✓** |
|  | Give the rationale for the prognosis |  |  | **✓** |

**5.** **RESPONDING TO PATIENT REACTIONS (n=24)**

|  | **BEHAVIOUR** | Literature | Consensus panel | Interviews with pwd |
| --- | --- | --- | --- | --- |
| *Explore the patient’s emotional response* | Do not anticipate the patient’s emotional reaction | **✓** |  |  |
|  | Acknowledge the difficulty of the issue | **✓** |  |  |
|  | Encourage articulation & disclosure of emotion | **✓** |  | **✓** |
|  | Observe the patient’s non-verbal reactions, acknowledge & check your interpretation of cues (e.g. ‘you are looking very worried’) | **✓** |  | **✓** |
|  | Show empathy – express personal concern for the patient’s distress & demonstrate your understanding of their feelings | **✓** |  | **✓** |
|  | Identify the reasons behind the emotional reaction | **✓** |  | **✓** |
|  | Actively manage emotional responses | **✓** | **✓** |  |
|  | Provide an opportunity for the patient to absorb & emotionally process the information | **✓** | **✓** | **✓** |
|  | Explore feelings of loss associated with the anticipated disability | **✓** |  | **✓** |
|  | Remember that the patient may use denial as a way of choosing not to know the diagnosis | **✓** |  | **✓** |
|  | Avoid giving advice, information or reassurance prematurely | **✓** |  |  |
| *Elicit & address patient questions & concerns* | Ask the patient what other information would be helpful | **✓** |  |  |
|  | Create time & space for the individual to explore what the diagnosis means to them | **✓** | **✓** | **✓** |
|  | Elicit patient concerns | **✓** | **✓** |  |
|  | Determine all patient concerns before exploring any in depth | **✓** |  |  |
|  | Validate patient concerns | **✓** |  |  |
|  | (Get patients to) prioritise problems so that they can be addressed in an appropriate order | **✓** |  |  |
|  | Explore patient concerns or questions to discover what the individual really believes & why they are asking the question | **✓** |  |  |
|  | Discuss patient questions on the same day | **✓** |  |  |
|  | Address fears & thoughts about the future | **✓** |  | **✓** |
|  | Recognise that the patient may shut down after hearing bad news & it may take some time before the patient can articulate the questions that are buzzing around in their head | **✓** |  | **✓** |
|  | Since patients may not know the appropriate questions or be hesitant to ask, anticipate critical unanswered questions & suggest discussing them | **✓** |  | **✓** |
|  | Answer questions fully | **✓** |  | **✓** |
|  | If unable to answer questions, explain why not |  |  | **✓** |

**6. FOCUSING ON QUALITY OF LIFE & WELL-BEING (n=17)**

|  | **BEHAVIOUR** | Literature | Consensus panel | Interviews with pwd |
| --- | --- | --- | --- | --- |
| *Foster hope* | Focus on realistic, rather than unrealistic, hope | **✓** | **✓** |  |
|  | Identify positive aspects to tell the patient | **✓** |  | **✓** |
|  | Emphasise preserved abilities & skills | **✓** |  | **✓** |
|  | Define patient strengths | **✓** |  |  |
|  | Emphasise quality of life | **✓** |  |  |
|  | Emphasise maintaining social activities | **✓** |  | **✓** |
|  | Focus on maintaining function | **✓** |  | **✓** |
|  | Help the patient to maintain a sense of self |  |  | **✓** |
|  | Enhance the patient’s self esteem & self-confidence | **✓** |  | **✓** |
| *Explore coping strategies* | Help the patient adapt to the news | **✓** |  | **✓** |
|  | Address the patient’s spiritual values & explore how s/he comes to terms with their suffering | **✓** |  |  |
|  | Acknowledge patient coping efforts | **✓** |  | **✓** |
|  | Elicit previous successful strategies used by pwd & family members members to cope with life events & encourage their use in the current situation | **✓** |  | **✓** |
|  | Facilitate family problem-solving regarding patient care | **✓** |  |  |
|  | Encourage openness with friends & family members about the changes that are taking place | **✓** |  |  |
|  | Ask about patient support systems / social context | **✓** |  |  |
|  | Provide an opportunity for the patient to gain some sense of mastery or control over the illness | **✓** |  | **✓** |

**7. PLANNING FOR THE FUTURE (n=41)**

|  | **BEHAVIOUR** | Literature | Consensus panel | Interviews with pwd |
| --- | --- | --- | --- | --- |
| *Clarify follow up arrangements* | Provide acute follow up to help patients talk about diagnosis & release emotions | **✓** |  |  |
|  | Provide an opportunity to discuss the diagnosis again, answer questions & clarify matters | **✓** | **✓** | **✓** |
|  | Encourage the patient to return with questions | **✓** |  | **✓** |
|  | Arrange regular follow up to facilitate monitoring of the patient’s progress integrating the diagnosis | **✓** |  | **✓** |
|  | Identify the future named key worker |  | **✓** | **✓** |
|  | Identify other ways for the patient to explore outstanding concerns in the near future | **✓** |  | **✓** |
|  | Ensure information is consistent across professionals | **✓** | **✓** | **✓** |
|  | Reassure patients that care will continue | **✓** |  |  |
| *Discuss support services available & the role of other agencies* | Identify further medical & social care pathways | **✓** | **✓** | **✓** |
|  | Provide (written) information on practical & emotional support available from health & social care services | **✓** | **✓** | **✓** |
|  | Provide (written) information on practical & emotional support available from relevant voluntary organisations | **✓** | **✓** | **✓** |
|  | Provide information on memory rehabilitation | **✓** |  | **✓** |
|  | Provide information on opportunities for peer support |  |  | **✓** |
|  | Document the information given & to whom | **✓** | **✓** | **✓** |
|  | Write to GP giving information (copy to pwd & family member) |  | **✓** | **✓** |
| *Negotiate management plan* | Identify a plan for what is to happen next | **✓** |  |  |
|  | Do not rush the patient to treatment | **✓** |  |  |
|  | Offer choice, encourage the patient to make choices & decisions to the level that they wish | **✓** |  |  |
|  | Leave serious decisions until the individual has had time to absorb the information | **✓** |  |  |
|  | Check clarity & acceptability of plan with patient | **✓** | **✓** |  |
|  | Check that patient concerns have been addressed | **✓** |  |  |
|  | Share power & control with patient, recognise that you are both ‘experts’ | **✓** |  | **✓** |
|  | Explain possible unexpected outcomes, what to do if plan is not working, when & how to seek help | **✓** |  |  |
|  | Encourage patient to be involved in implementing plans, to take responsibility & to be self reliant | **✓** |  |  |
|  | Individualise & target support to the patient & their family | **✓** |  |  |
|  | Identify the (practical) implications of the diagnosis | **✓** | **✓** | **✓** |
|  | Mention legal & financial issues, power of attorney, benefits & wills | **✓** |  | **✓** |
|  | Explore issues of capacity & consent | **✓** | **✓** |  |
|  | Disclose all the treatment options (including no action) | **✓** | **✓** | **✓** |
|  | Provide information on treatment offered (e.g. name, how it works, side effects, benefits & advantages) | **✓** |  | **✓** |
|  | Explain how drug treatment will be managed (efficacy assessment; NICE guidelines; withdrawal criteria) |  |  | **✓** |
|  | Explain reasons for treatment |  |  | **✓** |
|  | Address any differences of opinion with patients so that, together, you reach a conclusion that is acceptable & safe for the patient | **✓** |  | **✓** |
|  | Offer specific suggestions on how they might manage the day to day consequences of impairment | **✓** |  | **✓** |
| *Discuss prevention & health promotion* | Discuss the role of ill health, fatigue & the environment in exacerbating confused behaviour | **✓** |  | **✓** |
|  | Discuss how crises might be prevented | **✓** |  |  |
|  | Offer early psychosocial intervention to prevent future medical & social crises | **✓** |  |  |
|  | Emphasise the importance of prompt treatment of concurrent infections | **✓** |  |  |
|  | Discuss the importance of maintaining general health & nutrition | **✓** |  |  |
|  | Provide information on the interactive role of patient & family member well-being in crisis prevention | **✓** |  |  |
|  | Seek consent or assent for inclusion on any disease management or mental health registers |  | **✓** |  |

**8. COMMUNICATING EFFECTIVELY (n=51)**

|  | **BEHAVIOUR** | Literature | Consensus panel | Interviews with pwd |
| --- | --- | --- | --- | --- |
| *Develop rapport* | Maintain eye contact | **✓** |  |  |
|  | Use physical contact as appropriate | **✓** |  |  |
|  | Attract the patient’s attention before speaking | **✓** |  |  |
|  | Allay yourself with the patient through use of ‘we’ in describing management plans etc. | **✓** |  |  |
|  | Create an environment in which patients feel comfortable asking questions | **✓** |  | **✓** |
| *Use appropriate verbal & non-verbal communication* | Use simple language (active, declarative sentences) | **✓** |  |  |
|  | Avoid the over-use of pronouns | **✓** |  |  |
|  | Use high frequency common words | **✓** |  |  |
|  | Use the same language that the patient uses | **✓** |  |  |
|  | Ask concise, specific questions | **✓** |  |  |
|  | Check for understanding of yes/no questions |  |  | **✓** |
|  | Avoid euphemisms | **✓** |  | **✓** |
|  | Avoid the use of technical terminology or medical jargon | **✓** | **✓** | **✓** |
|  | Make intonation patterns obvious, clearly signal questions | **✓** |  |  |
|  | Avoid speaking too slowly (this increases the load on working memory) | **✓** |  |  |
|  | Repeat or reinforce information as required | **✓** | **✓** | **✓** |
|  | Consider providing a tape recording of the consultation | **✓** |  |  |
|  | Use pauses & syllables & word stress to highlight information | **✓** |  |  |
|  | Use gestures & facial expressions to convey information | **✓** |  |  |
|  | Adopt a non-confrontational style |  |  | **✓** |
|  | Avoid sending non-verbal messages that undermine the verbal message being conveyed | **✓** |  |  |
|  | Avoid body language that signals restlessness, lack of commitment or rejection | **✓** |  |  |
|  | Use visual methods of conveying information to aid comprehension & recall | **✓** |  | **✓** |
|  | ‘Walk’ patients through reading materials rather than leaving them to read the information on their own | **✓** |  | **✓** |
|  | Provide clear written information (on diagnosis) |  | **✓** | **✓** |
| *Use active listening skills* | Listen attentively | **✓** |  |  |
|  | Do not interrupt the patient, it is confusing & may cause them to forget what they were going to say | **✓** |  |  |
|  | Pay attention to the patient’s emotional expression as it may be more reliably meaningful than their words (respond to the message not the words) | **✓** |  |  |
|  | Attend to verbal & non-verbal cues | **✓** |  |  |
|  | Consider helping the patient with word-finding difficulties by suggesting the target word (‘Do you mean….?) | **✓** |  |  |
| *Involve the patient* | Leave space for patient to think before answering or to continue after pausing | **✓** |  |  |
|  | Facilitate patient responses verbally & non-verbally (e.g. encouragement, silence, paraphrasing, repetition) | **✓** |  |  |
|  | Check out patient statements which are vague or need amplification | **✓** |  |  |
|  | Periodically summarise to verify own understanding of what the patient has said | **✓** |  |  |
|  | Be patient-centred |  | **✓** |  |
|  | Do not talk over the patient | **✓** |  |  |
|  | Identify behaviours that are suggestive of unresolved concerns or expectations (e.g. reluctance to accept management plan) | **✓** |  |  |
|  | Address the patient directly |  |  | **✓** |
| *Structure & signpost the consultation* | Summarise at the end of each section before moving on | **✓** |  |  |
|  | Minimise or limit the number of topic changes | **✓** |  |  |
|  | Use transitional statements when moving onto a new area to signpost change of topic | **✓** |  |  |
|  | Structure interview in a logical sequence | **✓** |  |  |
|  | Do not impart too much information in one session | **✓** | **✓** | **✓** |
|  | Organise information into discrete chunks, develop a logical sequence | **✓** |  |  |
|  | Summarise the session briefly | **✓** |  | **✓** |
| *Consider issues of anti-discriminatory practice* | Show respect for the fundamental worth of all persons | **✓** |  | **✓** |
|  | Show respect for the values & beliefs of all cultural groups | **✓** |  |  |
|  | Be aware of personal values & cultural differences & how these might influence interactions | **✓** |  |  |
|  | Be aware of emotional reactions to patients | **✓** |  |  |
|  | Be aware of personal strengths & weaknesses in working with patients | **✓** |  |  |
|  | Arrange an interpreter if necessary (avoid using family members as translators) | **✓** | **✓** |  |
